# Do Neural Cells Communicate with Endothelial Cells via Secretory Exosomes and Microvesicles?

**DOI:** 10.1155/2009/383086

**Published:** 2009-08-03

**Authors:** Neil R. Smalheiser

**Affiliations:** Department of Psychiatry, UIC Psychiatric Institute MC912, University of Illinois at Chicago, 1601 W. Taylor Street, Chicago, IL 60612, USA

## Abstract

Neurons, glial, cells, and brain tumor cells tissues release small vesicles (secretory exosomes and microvesicles), which may represent a novel mechanism by which neuronal activity could influence angiogenesis within the embryonic and mature brain. If CNS-derived vesicles can enter the bloodstream as well, they may communicate with endothelial cells in the peripheral circulation and with cells concerned with immune surveillance.

## 1. Introduction

About a year and a half ago, I reviewed evidence that cells within the central nervous system may transfer vesicles containing RNAs and proteins among themselves in a novel type of cell-cell communication [1]. That paper emphasized the possible role of secretory exosomes as a mechanism providing dynamic activity-dependent transfer of molecules across synapses, corresponding to the morphological appearance of “synaptic spinules” which had been long noted by neuroanatomists. However, there are numerous additional examples in the nervous system of transfer of molecules by vesicles moving freely from cell to cell or by cytoplasmic “fingers” intruding directly from one cell into another. For example, astrocytes can provide neuroprotective HSP70 to neurons via exosomes [2], and Schwann cells can provide polyribosomes to the axons that they ensheath [3]. In this brief hypothesis paper, I point out the possibility that central nervous system-(CNS-) derived vesicles may potentially interact with endothelial cells within the brain, and that they may potentially find their way to the bloodstream, where they could interact with endothelial cells and with cells of the immune system. 

Secretory exosomes are formed by a specific process of invagination that occurs within endosomes, resulting in the formation of multivesicular bodies [4], or on the cell surface, resulting in budding-out from lipid raft regions of the plasma membrane [5]. Microvesicles are little fragments that are shed or pinched-off from the plasma membrane. Microvesicles are generally thought to be larger than exosomes, but their features and biogenesis may not be entirely distinct [6], and there may be additional types of vesicles that cannot be easily classified at present [7]. Exosomes and microvesicles have been shown to be shed in a regulated fashion by many cell types in culture, including neurons [8] and astrocytes [9]; they have cell-adhesion molecules on their surface which allow them to bind specifically to certain target cell types and to be internalized (e.g., [9, 10]). 

In several cases, the internalized mRNAs have been shown to be translated, suggesting that they provide a form of gene transfer to the target cells [9–11]. Studies of endothelial cells have shown that exosomes and/or microvesicles can alter their gene expression and activate thrombogenicity, apoptosis, and angiogenesis [11–15]. 

## 2. Do CNS-Derived Vesicles Interact with Endothelial Cells within the Brain?

Secretory exosomes have been detected within the cerebrospinal fluid both in the embryonic and mature brain [16, 17], and neuron-enriched microRNAs have been detected in the cerebrospinal fluid (CSF) as well [18]. This suggests that neural cells do release vesicles into the extracellular space in vivo. Vascularization and neurogenesis proceed concurrently within the developing brain [19, 20]; both involve similar events such as cell migration and differentiation, and both respond to some of the same patterning cues, growth factors (e.g., vascular endothelial growth factor), and so forth. Endothelial cells interact with neurons and glial cells to form a so-called functional “neurovascular unit” [21], and these interactions are necessary in order for endothelial cells to express tight junctions that underlie the blood-brain barrier [22]. Transfer of vesicles is potentially one way in which neural cells may interact with endothelial cells during embryogenesis. Moreover, new growth of blood vessels occurs in the mature brain and can be stimulated in response to neuronal activity (e.g., environmental enrichment [23]), another arena in which neural-derived cues interact with endothelial cells. Finally, Skog et al. (2008) have shown that glioblastoma-derived microvesicles can stimulate angiogenesis of brain capillary endothelial cells in vitro, a process that would be expected to support tumor growth in vivo [11].

## 3. Can CNS-Derived Vesicles Reach the Bloodstream?

Blood plasma or serum is an abundant source of microRNAs and mRNAs, which appear to be contained within secretory exosomes and/or microvesicles (e.g., [24–33]). Many different normal as well as tumor cell types contribute vesicles to the bloodstream. Placental-derived microRNAs have been shown to provide a biomarker of pregnancy [24], whereas vesicles bearing tumor-specific antigens have been shown to express microRNA profiles related to the tumor cells from which they derive (e.g., [25]). Acetaminophen overdose, which damages the liver as well as other organs, results in elevated levels of the liver-specific microRNA mir-122 [33]. 

To date, no evidence has been published demonstrating that vesicles shed by CNS neurons or glial cells can enter the bloodstream. (Glioblastoma cells have been reported to shed vesicles into the blood [11], but their relation to nearby blood vessels may be aberrant and not representative of normal glial cells.) However, acetaminophen overdose causes elevated levels of numerous microRNAs in the blood that are generally thought to be brain-enriched [33]. This was interpreted by the authors as likely due to neural damage produced by the drug. Moreover, Dr. Samuil Umansky, Chief Scientific Officer of Xenomics, Inc., presented unpublished data at the Cambridge Healthtech Institute conference on “microRNA in Human Disease and Development” in Boston, MA, in March 2009, showing that microRNAs characteristics of brain expression were detectable in human blood and urine. Levels of these microRNAs were elevated in individuals poststroke in a time-dependent manner and were elevated in individuals diagnosed with Alzheimer disease, though it was not examined whether the microRNAs were contained within vesicles. 

What mechanisms might permit CNS-derived vesicles to reach the bloodstream? The blood-brain barrier is thought to prevent movement of large molecules into and out of the brain, and it is unlikely that vesicles would be actively transported across capillaries. However, the blood-brain barrier appears gradually during gestation [34] and so exosomes may be free to communicate with the blood at developmental stages. In the mature brain, it is conceivable that clearance of the cerebrospinal fluid into the blood may permit exit of intact vesicles. As well, the circumventricular regions of the brain appear to be devoid of a blood-brain barrier (including the pineal gland, area postrema, choroid plexus, subfornical organ, supraoptic crest, median eminence, and posterior pituitary) [35]. Furthermore, exit of vesicles may be expected to occur under pathological conditions in which the blood-brain barrier is compromised, for example, following trauma, cell death, or inflammation.

## 4. Conclusion

There is a growing appreciation that secretory exosomes, microvesicles, and possibly other types of cell-derived vesicles comprise a physiological channel for cell-cell communication, both among neighboring cells and within the bloodstream. Neurons and glial cells in the brain also appear to shed vesicles that potentially may contribute to trophic interactions and synaptic plasticity [1]. CNS-derived secretory exosomes and/or microvesicles have the potential to interact with endothelial cells during developmental stages and during angiogenesis within the mature brain. These interactions should have functional significance, insofar as neurogenesis and angiogenesis are, in part, coordinated responses both in the developing and mature brain [20, 36, 37]. 

Recent studies also raise the possibility that CNS-derived vesicles may enter the bloodstream and interact with endothelial cells in the peripheral circulation. This would represent a novel communication channel between the nervous system and the cardiovascular system. Circulating vesicles also appear to have an important role in immune surveillance and activation [7]. Perhaps future issues of *Cardiovascular Psychiatry and Neurology* will contain articles that provide evidence for this channel and that explore the meaning of its messages.
